# Visual attention and response time to distinguish athletes from non-athletes: A virtual reality study

**DOI:** 10.1371/journal.pone.0324159

**Published:** 2025-05-23

**Authors:** Lucia Imperiali, Stefano Borghi, Sara Bizzozero, Elena Prandoni, Ambra Bisio, Antonio La Torre, Roberto Codella

**Affiliations:** 1 IRCCS Istituto Ortopedico Galeazzi, Milan, Italy; 2 Department of Biomedical Sciences for Health, Università degli Studi di Milano, Milan, Italy; 3 Centro Polifunzionale di Scienze Motorie, Università degli Studi di Genova, Genoa, Italy; 4 Department of Experimental Medicine, Section of Human Physiology, Università degli Studi di Genova, Genoa, Italy; 5 Department of Endocrinology, Nutrition and Metabolic Diseases, IRCCS MultiMedica, Milan, Italy; The University of Tennessee at Chattanooga, UNITED STATES OF AMERICA

## Abstract

In recent years, cutting-edge technologies have been increasingly integrated into sports to assess physical and cognitive capacities. Virtual Reality (VR) technology has emerged as a powerful tool, offering an immersive and controlled scenario for examining cognitive functions. In light of the growing adoption of VR-systems, this study aimed to investigate the differences in visual attention and response time (RT) between athletes and non-athletes utilizing a VR-system and evaluate the discriminatory power of VR assessments towards the two groups. Sixty-one participants (Age: 22 ± 1.8years; athletes = 33, non-athletes = 28) underwent two visual attention evaluations through the Multiple Object Tracking (MOT) paradigm and three RT evaluations, all within a fully immersive VR environment. The visual attention assessments included the “MOT Assessment” (MOT), which did not have a primary target to select, and the “MOT, Primary Target Onset” (MOT-PT), which required selecting a primary target. The three RT evaluations included Continuous RT (RT-C), RT with Inter-Time between stimuli (RT-I), and Go No-Go RT (RT-GNG). In all RT assessments, participants aimed to touch the target that turned green in the shortest time as possible. In the RT-GNG test, participants responded to green targets by touching them with the hand controllers and refrained from touching red targets. No differences were found between athletes and non-athletes in visual attention tasks. However, athletes outperformed non-athletes in RT assessments (RT-C: 504.8 ± 45.9ms vs. 549.1 ± 45.6ms; p < 0.001. RT-I: 481.1 ± 44.9ms vs. 534.2 ± 58.6ms; p < 0.001. RT-GNG: 502.9 ± 38.8ms vs. 555.6 ± 57.8ms; p < 0.001). ROC curve analysis demonstrated moderate accuracy in differentiating athletes from non-athletes in RT assessments (RT-C: AUC = 0.75, p < 0.001; RT-I: AUC = 0.75, p < 0.001; RT-GNG: AUC = 0.80, p < 0.001). These findings underscore the significant role of RT in distinguishing athletes from non-athletes and highlight the discriminative potential of VR-systems as valuable tools in sports evaluation. Including RT assessments into traditional training regimens could offer new insights for evaluating athletic performance.

## Introduction

In many daily contexts, such as driving, work tasks, and sports activities, individuals are challenged to visually perceive and cognitively process multiple moving objects and scenes within a short amount of time [1]. For this reason, visual attention and time to response are critical processes for making accurate decisions, reflecting the efficiency of cognitive processing and motor execution [2,3]. Visual attention is a process that allows for the selection of certain stimuli while ignoring others, facilitating the cognitive organization of appropriate actions in response to relevant stimuli [4]. Thus, it can be considered a sub-process of perception that filters the vast array of available information to highlight aspects relevant to the organism [1]. On the other hand, response time (RT) refers to the duration of the motor response to a visual stimulus, measuring the time from stimulus onset to the completion of the movement [5]. Therefore, both of these processes are essential in everyday life and even more in sports, particularly in open skill sports (e.g., basketball, tennis, squash, or boxing) where participants must respond and adjust to an unpredictable and rapidly changing environment [6]. Similarly, in team sports, maintaining a high level of attention during the game enables players to recognize changes in teammates and opponents’ positions and simultaneously track the ball dynamically. For this reason, a high level of visual attention and fast RT allow for achieving better performance [7,8]. It is well known that an individual’s cognitive abilities, including working memory, processing speed, and decision-making, directly affects visual attention [9]. Athletes typically develop enhanced cognitive processing skills through training, enabling them to focus on relevant visual stimuli while ignoring distractions [10–12]. This ability is crucial in sports, where split-second decisions can determine the outcome of a play. While visual attention is important across sports, previous studies have rarely compared athletes from different disciplines [13], likely due to the unique cognitive and perceptual demands each sport imposes [14].

In recent years, new technologies have been used in sports as innovative tools for assessment of physical capacities, mental training and rehabilitation [15,16]. The advent of Virtual Reality (VR), an immersive technology which enables users to interact with 3D computer generated simulation of a real environment, in real time, using their senses and motor skills, has provided a novel platform for studying cognitive and physical functions [17]. VR allows for the simulation of realistic scenarios, enhancing ecological validity while maintaining experimental control [18]. Generally, VR can be categorized by presentation methods into non-immersive systems (typically displayed on a computer screen) and immersive systems (such as those using headsets and cave environments) [19]. Non-immersive VR-systems are more cost-effective but are better suited for experiments requiring simple reactions due to their less immersive nature [20]. In contrast, immersive VR-systems provide a more realistic and complex physiological and psychological experience, which has become the main focus of VR research and applications in recent years [21]. VR is a widespread technology drawing an increasing interest for athletes and coaches, as it offers a tool to simulate, analyse and train situations that are often too complex to reproduce in the field. Using VR, there is the possibility of adding or removing elements from the immersive scenario, stratifying the reality that the athlete has to deal with to make exercises more basic or more complex [16]. Replicating this kind of scenario in the real world is very difficult, and in some cases, impossible.

To date, most of the research on visual attention and RT has employed visual displays with 2D sets [22]. However, traditional video simulations on computer screens offer only modest levels of immersion in the action [23]. In addition, these simulations are not sufficiently controllable to allow an objective assessment, nor are they complex enough to reflect the challenges analogous to those encountered by people in their daily lives [22]. Given the increasing adoption of VR systems, this study introduces a novel approach that aligns neuropsychological assessment with ecological tests by simulating real-life scenarios while ensuring reproducibility and controlled regulation of test conditions [24]. In detail, the study leverages an advance VR system to examine differences in visual attention and RT between athletes and non-athletes. Unlike previous studies, our study integrates a fully immersive VR environment, offering a more realistic and dynamic setting of evaluation. This allows for a more precise appraisal of cognitive and perceptual abilities under conditions that closely mirror real-world demands. Furthermore, the study evaluated the discriminatory power of VR-based assessments in distinguishing between the two groups. We hypothesized that athletes would outperform non-athletes in both visual attention and RT assessments.

## Materials and methods

### Study design

This observational, cross-sectional study was conducted at the Università degli Studi di Milano (Milan, Italy) between January 15, 2024, and May 17, 2024. The study protocol was approved by the Ethics Committee of the university (ref. n.: 126/23) and adhered to the ethical principles for medical research involving human participants as outlined in the latest version of the World Medical Association Declaration of Helsinki. All participants were informed about the purpose, methods, potential risks, and benefits of the study and provided written informed consent. All participants completed the following evaluations: i) Assessment of visual attention using a VR-system (VR-Brain Tracker software); ii) Assessment of RT using a VR-system (VR-CNS Sprint software); iii) A survey collecting demographic data, sport practice information, and details on the duration and frequency of training. Before conducting the data collection tests, a familiarization session with the VR-system was performed. This session aimed to ensure that all participants were comfortable and accustomed to the VR environment and its interface. During this phase, participants had the opportunity to explore the system, learn how to interact with the virtual scenario, and understand the protocol of the VR assessments. Each subject completed all the tests within their usual training time. Based on survey responses, the sample was categorized into two groups: athletes and non-athletes. Participants were classified as active/trained, following McKay classification [25]. Active participants (Tier ≥ 2) were registered with Italian sports federations. Sedentary participants (Tier 0) either exhibited sedentary behaviours or trained less than two hours per week, and were not registered with any Italian sports federations.

### Participants

Participants were enrolled at the Università degli Studi di Milano (Milan, Italy). The subjects completed all the assessments within a single day. Participants were engaged for approximately 40 minutes in total, including the time required for survey completion, as well as the visual attention and RT evaluations. The inclusion criteria for selecting participants included: male and female students; aged between 18 and 30 years; classified sedentary (tier 0) or active (tier ≥ 2) following McKay classification [25]; no experience with VR-systems. The exclusion criteria were as follows: not providing informed consent for the study; neurological or orthopaedic pathological conditions that could potentially affect movement, vision conditions, pathologies or use of medication that could affect the correct execution of assessments. We considered 15 team sport athletes and 18 individual sport athletes. Specifically, the sports included in the study were: Track and Field (7 athletes), Basketball (2 athletes), Beach Volleyball (1 athlete), Climbing (1 athlete), Combat Sports (2 athletes), Dance (2 athletes), Flag Football (1 athlete), Gymnastics (1 athlete), Rowing (5 athletes), Football (1 athlete), Volleyball (6 athletes), and Water Polo (4 athletes). All participants classified as athletes had a minimum of seven years of experience in their respective sports (mean sport experience: 13.1 ± 5.8 years).

### Virtual reality system

The VR-system used comprises four components: a computer, a headset, two controllers, and specialized software (VR-Brain Tracker and VR-CNS Sprint). The Meta Quest 2 headset (Meta, California, USA) delivers an immersive 360° viewing experience, offering high-resolution imagery at 20 pixels per degree and employing a rapid-switching LCD display boasting 1832 x 1920 pixels per eye. It is equipped with two controllers for precise hand tracking. The headset is powered by 6 GB of RAM integrated with a Qualcomm Snapdragon XR2 platform, ensuring smooth performance. Additionally, it features built-in native 3D audio capabilities. The computer was a Lenovo IdeaPad Gaming 3 15IAH7 model (Lenovo, Beijing, China) with Intel® Core™ i7-12650H processor with 16 GB RAM, 15.56-inch 1920 x 1080-pixel screen and Microsoft Windows 11 Home operating system with Nvidia RTX 3060 graphics card. The two softwares used in the study were developed and provided by Mind Room Lab s.r.l. (Bassano del Grappa, Italy).

### VR-Brain Tracker

**Fig 1:**
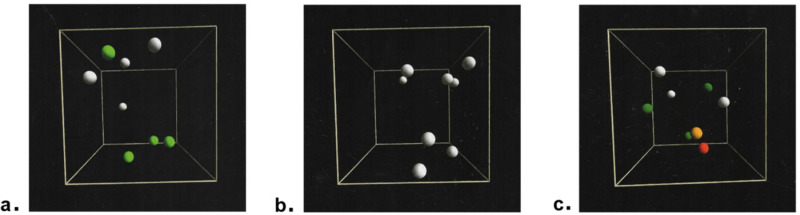
Virtual room of MOT performed by VR-Brain Tracker software. a) Identification of targets coloured in green; b) Movement of the 8 spheres in the cube; c) Selection of targets.

VR-Brain Tracker software assessed visual attention and memory skills through two evaluations: i) Multiple Object Tracking (MOT); ii) Multiple Object Tracking with Primary Target Onset (MOT-PT). The MOT comprised 20 repetitions within a VR cube, where 8 spheres were displayed. During each repetition, 4 of these spheres randomly flashed green for 3 seconds (Fig 1A), after which all spheres returned to their original colour and begin moving in various directions within the cube for 7 seconds (Fig 1B). Subsequently, the spheres come to a halt, prompting the participant to identify the 4 spheres initially highlighted in green (Fig 1C).

Successful selections prompted an increase in the movement speed of spheres in the next repetition and in the final score of the assessments, while errors lead to a decrease in their movement speed (Fig 1C); red sphere represents the incorrect selection, whereas the orange sphere indicates the unselected correct target. Upon completion, the system released an Attentional Index (AI), starting from a baseline score of 1.2. Higher scores in AI indicate better visual attention. MOT-PT differed from MOT by including one primary target and two secondary targets, which were distinguished by their colours: blue for the primary target and green for the secondary targets. In each repetition, the primary target was the first one the participant had to select. The AI for MOT (AI_MOT_) and MOT-PT (AI_MOT-PT_) tests were calculated using the Staircase method [26], an adaptive method where each correct response leads to a decrease in the signal level, while each incorrect response increases it. The Staircase method is used to keep stimuli close to the threshold value, adapting the sequence of presentation in relation to the subject’s response, reversing the tendency at each change in the response itself [27]. Unlike the traditional up-down method, the Staircase method utilizes different step sizes for upward and downward adjustments. In our study, we employed 1-step increase and − 1.5-step decrease. The test was conducted with the participant seated on a chair. They remained stationary during the evaluations and did not move around the VR-environment. Participants made their decisions using hand controller.

### VR-CNS sprint

**Fig 2:**
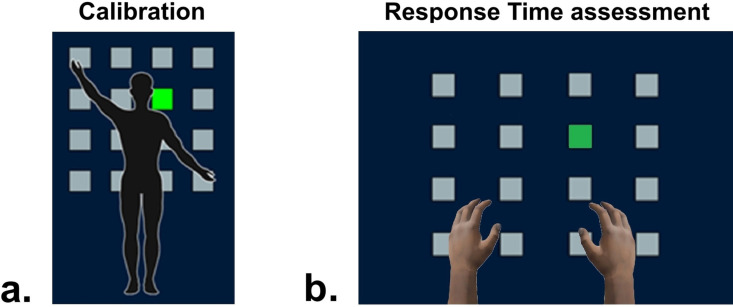
Virtual room of RT assessments performed by VR-CNS Sprint software. a) Adjustment of targets height and distance; b) Virtual room during assessments.

VR-CNS Sprint assessed RT in response to visual targets appearing randomly in the field of view (Fig 2).

The assessments were always conducted using a standard panel with targets positioned within the visual field. The panel consisted of 16 targets arranged in 4 rows and 4 columns. The size of the panel was chosen based on the participant’s arm reach, with three options available: extra small (70x70 cm), small (90x90 cm), and medium (120x120 cm). For this study, we consistently used the 70x70 cm panel.

Participants wore hand controllers and were instructed to touch the target that turned green as quickly as possible (hit). The evaluation comprised three tests: i) RT in Continuous (RT-C); ii) RT with Inter-time between stimuli (RT-I); iii) RT with inter-time between stimuli Go No-Go paradigm (RT-GNG). In RT-C test, targets sequentially turned green in random order immediately after the participant previous hit. The evaluation included a total of 96 hits, with each target being activated six times (16 targets x 6 = 96 hits). RT-I test was characterized from the introduction of intervals of rest between stimuli (0.25s, 0.50s, 0.75s or 1.00s). The interval of rest was randomly repeated between hits, and the assessment included a total of 96 hits, with each target being activated six times (16 targets x 6 = 96 hits). Finally, in RT-GNG, RT was assessed using the Go No-Go paradigm [28]. Target could appear either green (Go) or red (No-Go). Rest intervals of 0.25s, 0.50s, 0.75s or 1.00s was randomly repeated between targets appearance. The No-Go targets remained illuminated in red for 2.0s before automatically turning off. Participants were instructed to not touch No-Go targets and no errors were performed during the experimental procedures. The distribution included 96 Go targets and 32 No-Go targets (Go targets: 16 targets x 6 = 96 hits; No-Go targets: 16 targets x 2 = 32).

### Statistical analysis

Variables were expressed as mean ± standard deviation (SD) and 95% confidence intervals (CI). Based on literature research, to determine the sample size *a priori* (software package, G*Power 3.1.9.2), the following input variables were selected as per a t-test analysis with two tails: a statistical power (1-β) of 0.90, a probability α level of 0.05, an effect size (Cohen *d*) of 0.937, two groups. These inputs were determined by considering RT as primary outcome. Using these parameters, a total sample size of 50 subjects is obtained (25 subjects for each group). To account for any potential subject drop-out during the scientific procedures (which should not exceed 10%), the sample size was increased to 28 subjects per group. The normality of the distribution of the outcome measures was checked using graphical methods and the Shapiro–Wilk test. In order to assess differences between sport practice (athletes vs non-athletes), the Mann-Whitney test was performed in case of non-normality of data (age, AI_MOT-PT_, RT-GNG). For data with normal distribution, the unpaired t-test was used. Effect sizes for pairwise comparison were calculated using Cohen’s *d* and categorized as trivial (effect size: < 0.20), small (0.21–0.60), moderate (0.61–1.20), large (1.21–2.00), or very large (> 2.00) [29]. The significance level was set at p < 0.05. The receiver operating characteristics (ROC) curve analysis was performed to ascertain the accuracy and validity of this VR battery assessment. The area under the curve (AUC) value was analysed to determine the discriminatory power of VR assessments towards athletes and non-athletes: high accuracy (AUC > 0.9), moderate accuracy (AUC 0.7 to 0.9), low accuracy (AUC 0.5 to 0.7) or a chance result (AUC < 0.5) [30,31]. Statistical analysis was performed using Graph Pad Prism Software, version 8.0 for Windows (Graph Pad Software, San Diego, CA).

## Results

### Study population

A total of 88 students were screened for eligibility and 61 young adults were recruited. The sample (males: n = 32; females: n = 29) included 28 non-athletes and 33 athletes, aged between 19 and 26 years (total sample: 22.6 ± 1.9 years. Non-athletes: 22.6 ± 2.1 years, Athletes: 22.6 ± 1.7 years; p = 0.687).

### VR-Brain Tracker and VR-CNS sprint

#### Athletes and non-athletes.

Table 1 reports the results obtained by comparing athletes and non-athletes with the two dependent variables (visual attention and RT).

**Table 1:** Comparison between athletes and non-athletes in VR-Brain Tracker and VR-CNS Sprint assessments.

Variable	Total sample (n = 61)	Athletes (n = 33)	Non-athletes (n = 28)	p (ES)
**AI**_**MOT**_ (au)	3.0 ± 1.0 (2.7–3.3)	3.1 ± 1.1 (2.7–3.4)	2.9 ± 1.0 (2.5–3.3)	0.605
**AI**__**MOT**__**_-_**__**PT**__ (au)	3.6 ± 0.9 (3.3–3.8)	3.6 ± 0.9 (3.3–3.9)	3.5 ± 1.0 (3.1–3.9)	0.980
**RT-C** (ms)	525.2 ± 50.5 (512.2–538.1)	504.8 ± 45.9 (488.6–521.1)	549.1 ± 45.6 (531.5–566.8)	**<0.001** **(0.97)**
**RT-I** (ms)	505.5 ± 57.7 (490.7–520.3)	481.1 ± 44.9 (465.2–497.0)	534.2 ± 58.6 (511.5–556.9)	**<0.001** **(1.03)**
**RT-GNG** (ms)	527.1 ± 54.8 (513.0–541.1)	502.9 ± 38.8 (489.1–516.6)	555.6 ± 57.8 (533.2–578.0)	**<0.001** **(1.09)**

Data are reported as mean ± SD (lower and upper 95%CI). AI_MOT_: Attentional Index of the MOT; au: arbitrary units; AI_MOT-PT_: Attentional Index of the MOT-Primary Target Onset; ES: Effect size; p: p-value; RT-C: Response Time in Continuous; RT-GNG: Response Time with Inter-time between Stimuli, Go No-Go paradigm; RT-I: Response Time with Inter-time between Stimuli.

ROC results are shown in Table 2. In detail, all RT assessments detected a moderate level of accuracy considering the AUC. RT-GNG showed the highest AUC (AUC = 0.80, p < 0.001) as compared to all other ROC results, followed by RT-I (AUC = 0.75, p < 0.001) and RT-C (AUC = 0.75, p < 0.001). Low accuracy was detected in visual attention assessments (MOT: AUC = 0.54, p = 0.572; MOT-PT: AUC = 0.50, p = 0.977).

**Table 2:** ROC results for the MOT, MOT-PT, RT-C, RT-I and RT-GNG conducted in athletes and non-athletes.

Variable	AUC	p value	Sensitivity (%)	Specificity (%)	Cut - point
**AI**_**MOT**_ (au)	0.54	0.572	82.14	33.33	3.77
**AI**__**MOT**__**_-_**__**PT**__ (au)	0.50	0.977	17.86	96.97	2.18
**RT-C** (ms)	0.75	<0.001	78.57	63.64	521.50
**RT-I** (ms)	0.75	<0.001	71.43	78.79	513.50
**RT-GNG** (ms)	0.80	<0.001	75.00	78.79	531.50

AI_MOT_: Attentional Index of the MOT; AI_MOT-PT_: Attentional Index of the MOT-Primary Target Onset; au: arbitrary units; AUC: Area Under the Curve; RT-C: Response Time in Continuous; RT-GNG: Response Time with Inter-time between Stimuli, Go No-Go paradigm; RT-I: Response Time with Inter-time between Stimuli.

## Discussion

Based on the current literature, this study is among the first to analyse differences in visual attention and RT between athletes and non-athletes using VR applications. The main findings of this study were that athletes showed faster RT than non-athletes, and ROC curve analysis showed moderate accuracy in distinguishing athletes from non-athletes in RT assessments.

The present study did not detect significant differences in AI_MOT_ and AI_MOT__-___PT__ scores between athletes and non-athletes. Attention and performance in athletes have been widely studied using the MOT paradigm, as evidenced by previous research [32,33]. Nonetheless, findings regarding the performance advantages of athletes remain inconsistent. For example, several studies have reported that athletes outperform non-athletes in MOT tasks [32–34]. However, other studies have presented contrasting results. Memmert and colleagues [8], found that experts in team sports did not perform better on basic attention tasks than athletes from individual sports or novice athletes. Similarly, a meta-analysis by Liu et al. [33] observed that when the number of tracking targets was limited, there was no significant difference in tracking accuracy between expert athletes and novices. Additionally, one study within the analysis reported that basketball players had lower MOT scores compared to non-athletes.

These contrasting results potentially due to various factors influencing differential performance in MOT tasks. For example, the difficulty level of the MOT task can be varied using different number of targets [35] and distractors [36], the speed of the targets [37], and the duration of tracking [38]. Furthermore, attentional capacities, such as selective attention [39], sustained attention [40], and divided attention [41], can be influenced by various factors including the type of sport, level of expertise, and even the individual’s physiological state [42,43]. Additionally, various factors, such as certain daily life activities, could help explain the differences observed in MOT performance. For example, gamers often possess greater familiarity with technology, which may enhance their visual attention skills and, consequently, their MOT abilities, due to the consistent engagement with video games [44]. The topic of attention in athletes is widely debated, and undoubtedly requires further and more in-depth research.

Comparing the results from RT assessments, athletes showed faster RT than non-athletes. These findings align with existing literature, which consistently shows that athletic training and sport practice positively impacts RT performance [7,45]. RT is a crucial measure in sports performance, as it reflects the speed of information processing and the ability to execute motor responses quickly and accurately [6]. Faster RT can provide a competitive advantage, particularly in sports that require rapid responses to changing stimuli. The most common methods in the literature to assess RT in athletes are through the use of computer [6,45,46]. In the study of Akarsu and colleagues [45], the RT of athletes was compared to that of non-athletes. Their results support the view that sport activities and training are beneficial for RT, as athletes performed better than non-athletes in the study assessments. In support of this, Hülsdünker and colleagues [7] explained that the faster visuomotor RT observed in athletes, compared to non-athletes, may be attributable to both structural and functional adaptations in the central nervous system. Structural adaptations, such as increased myelination and enhanced synaptic plasticity, contribute to faster signal transmission in the nervous system [47]. Additionally, functional changes, including improved coordination between sensory input and motor output, play a pivotal role in enhancing RT [48]. Specifically, chronic training seems to induce adaptations in visual and motor-related processes, affecting both neural function and the structure of grey and white matter [49,50]. These structural and functional adaptations not only explain the enhanced visuomotor RT in athletes but also highlight the broader implications of neuroplasticity in sports training and its potential benefits for individuals at all levels of physical activity [51]. Neuroplasticity refers to the brain’s ability to modify its structure and function in response to the stimuli it encounters [52,53]. These neural adaptations are not limited to elite athletes but can also be observed in individuals engaging in regular physical activity, suggesting that even moderate exercise can enhance cognitive and motor functions [54].

The ROC analysis supported the discriminative validity of the VR setup, as the results showed moderate accuracy in the AUC values for RT assessments between athletes and non-athletes. In fact, an AUC between 0.75 and 0.80 indicates that RT assessments can be classified as a fair, albeit not exclusive, screening tool to distinguish athletes from non-athletes.

The limitations of this study include the reliance on a questionnaire that only investigated the type of sport practiced and the minutes spent training, without incorporating a training diary to calculate the training load more comprehensively. Additionally, the equipment used in the study lacked specificity to the sports being analysed, which may have influenced the precision and applicability of the findings. In addition, the use of a VR-system offers several advantages over traditional 2D technologies, such as computers [55], that could help overcome some of these limitations in future studies. In our study we try to better understand attentional aspect in athletes using VR technology. This technology allowed for a 3D-MOT, which has been shown to offer several advantages compared to 2D-MOT, such as creating and controlling virtual motion scenes [56], presenting stereoscopic vision [57] and immersing the visual scene. Based on these advantages, some studies indicated that VR technology can more effectively measure perception and motor performance in the field of motion than traditional methods [55]. For this reason, the setup used in the study could be considered a novel approach to evaluate attention and RT in a more lifelike virtual space. This opens up exciting possibilities for practical applications in sports training, benefiting both coaches and sport scientists. Athletes can leverage this technology to enhance their cognitive skills, improve RT, and sharpen focused attention − key factors for excelling in competitive sports. These benefits extend across various disciplines, including from team and individual ball sports, combat sports, motorsports and racing, precision and shooting sports, as well as water and endurance sports.

## Conclusions

This study utilized VR to assess visual attention and RT and explore how these processes are influenced by sport practice. The findings suggest that athletes exhibit faster RT compared to non-athletes, highlighting that physical training not only enhances motor skills and physical performance but also positively affects information processing speed. This insight could lead to the development of training programs that integrate cognitive exercises, such as visual attention and RT tasks, to further enhance athletes’ performance. Additionally, VR-based assessments show promise as screening tools for athlete selection, providing a more comprehensive evaluation of cognitive abilities, including visual attention and RT, alongside physical skills. By increasing immersion through a VR scenario, individuals may experience greater engagement and emotional connection, making the training feel more realistic and impactful.

Future research should investigate the effectiveness of VR as a training tool by comparing it with existing methodologies and determining whether a VR-based training with can improve visual attention and RT in athletes.
